# Rapid Detection of Clarithromycin and Amikacin Resistance in Mycobacterium abscessus Complex by High-Resolution Melting Curve Analysis

**DOI:** 10.1128/spectrum.00574-22

**Published:** 2022-05-31

**Authors:** Haoran Li, Gulibike Mulati, Yuanyuan Shang, Cong Yao, Yufeng Wang, Weicong Ren, Zhongtan Xue, Shanshan Li, Yu Pang

**Affiliations:** a Department of Bacteriology and Immunology, Beijing Chest Hospital, Capital Medical University/Beijing Tuberculosis and Thoracic Tumor Research Institute, Tongzhou District, Beijing, People’s Republic of China; b Department of Laboratory Medicine, The Eighth Affiliated Hospital of Xinjiang Medical University Urumqi, Xinjiang, People's Republic of China; c Department of Laboratory Quality Control, Innovation Alliance on Tuberculosis Diagnosis and Treatment (Beijing), Beijing, People’s Republic of China; Louisiana State University Health Sciences Center

**Keywords:** *Mycobacterium abscessus* complex, clarithromycin, amikacin, resistance, melting curve analysis

## Abstract

The emergence of Mycobacterium abscessus complex (MABC) infection is the most noteworthy health care problem. Clarithromycin (CLA) and amikacin (AMK) constitute the cornerstone of treatment for patients infected with MABC; thus, early detection of resistance to these two drugs is essential for formulating effective therapeutic regimens. In the present study, we aimed to validate the use of MeltPro MAB assay, a melting curve analysis with dually labeled probes, on a set of clinical isolates to detect CLA and AMK resistance. A total of 103 clinical MABC strains were collected in our analysis, including 76 strains of M. abscessus *subsp*. *Abscessus* (MAA) and 27 strains of M. abscessus *subsp. Massiliense* (MAM). *In vitro* susceptibility testing revealed that two isolates exhibited intrinsic CLA resistance by harboring A2270T mutation in *rrl*, and inducible resistance was noted in 42 isolates. Additionally, two MAA isolates with erm(41)T28 genotype were susceptible to CLA. Notably, we found three out of 44 isolates had two melting curve peaks, representing the simultaneous presence of mutant and the wild type in these specimens. In contrast, no known mutations were identified in six AMK-resistant isolates. Further analysis revealed that MeltPro yielded 100% and 96.67% sensitivity and specificity for detecting CLA resistance. In summary, this study firstly demonstrates that MeltPro is a promising diagnostic for early detection of CLA resistance for MABC isolates, which significantly improves the turnaround time within 2 h. Approximate two fifths of MABC isolates are resistant to CLA by 23S rRNA mutation or its methylation, emphasizing the urgent need for early detection of CLA resistance prior to empirical treatment of MABC infections.

**IMPORTANCE** Mycobacterium abscessus complex (MABC) has attracted increasing attention due to the numerous cases of infection. This pathogen is notorious for its intrinsic drug resistance, which complicates clinical management of patients with MABC infections. Clarithromycin (CLA) and amikacin (AMK) are the cornerstone of treatment regimens for MABC. Herein, our data firstly demonstrates that MeltPro is a promising diagnostic for early detection of CLA resistance for MABC isolates. The high frequency of CLA-resistant MABC isolates in China emphasizes the urgent need for early detection of CLA resistance prior to empirical treatment of MABC infections.

## INTRODUCTION

Although Mycobacterium tuberculosis remains one of the most significant public health threats, many other species of mycobacteria called nontuberculous mycobacteria (NTM) have attracted increasing attention due to the numerous cases of infection worldwide, especially in low tuberculosis incidence settings (1, 2). NTM are common in a variety of environmental niches, such as food, water, soil, and animals (3). As opportunistic pathogens, NTM infections occur most frequently in immunocompromised hosts and individuals with structural lung diseases (4).

NTM can be divided into rapid-growing mycobacteria (RGM) and slow-growing mycobacteria (SGM) according to their growth rate, with most NTM species belonging to the RGM (5). Mycobacterium abscessus complex (MABC) is the most prevalent RGM to cause human diseases. This pathogen is notorious for its intrinsic drug resistance, which complicates the clinical management of patients with MABC infections (6). The mobility rate of susceptible population has been close to 20% in the past few decades (7). The therapeutic effect of MABC is mainly limited to the fact that MABC is insensitive to “standard” antituberculosis drugs defined by the World Health Organization (WHO) and is only sensitive to limited antibiotics such as AMK and macrolides. Macrolides and amikacin (AMK) constitute the cornerstone of treatment for patients infected with MABC (8); therefore, early detection of resistance to these two antibiotics is important for formulating effective therapeutic regimens for the patients in view of the significant diversity in drug susceptibilities of the MABC isolates (8, 9).

Spontaneous mutations in the key antibiotic targets are identified to confer drug resistance in mycobacterial species. In M. abscessus, the nucleotide alternation in 16S rRNA (*rrs*) is associated with resistance to aminoglycosides (10). Resistance to clarithromycin (CLA) harbored by a mutation in the 23S rRNA (*rrl*) generally occurs in M. abscessus (11, 12). Another resistance mechanism developed by M. abscessus to CLA is an inducible resistance conferred by *erm*(41), encoding an erythromycin ribosomal methylase gene (13). Increasing evidence shows that the mutations associated with CLA and AMK are the promising predictive biomarkers of drug resistance (14, 15). However, no commercial kits are available for molecular identification of CLA and AMK resistance of MABC isolates.

**FIG 1:**
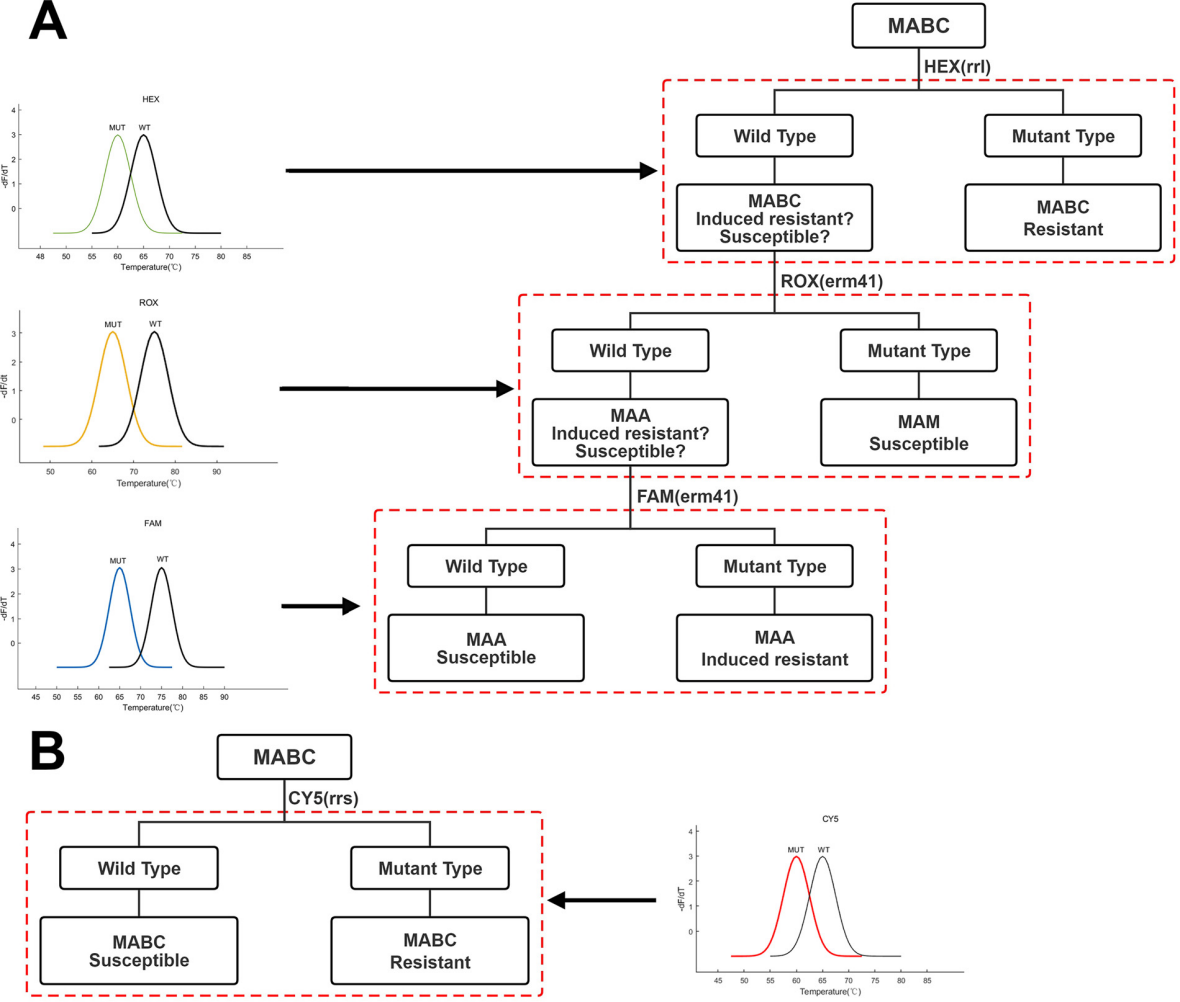
Decision tree diagram based on tandem MeltPro MAB assay. (A) The MAM CLA sensitivity, MAA CLA sensitivity, and MAA CLA resistance in MABC can be distinguished with high-resolution by the melting curves produced by HEX, ROX, and FAM. (B) Through the melting curve produced by CY5, the AMK sensitivity and AMK resistance of MABC can be distinguished with high-resolution.

Recently, a novel MeltPro MAB assay (MeltPro) (Zeesan Biotech, Xiamen, China) has been developed for the detection of resistance to CLA and AMK using melting curve analysis with dually labeled probes, which holds considerable promise for detecting drug-resistant MABC (Fig. 1). In the present study, we aimed to validate the use of the MeltPro MAB assay on a set of clinical isolates for the detection of CLA and AMK resistance. The results provide insight into the potential to scale up this new diagnostic in China.

## RESULTS

### MIC result of MABC strains.

A total of 103 clinical MABC strains were collected from the China Nontuberculous Mycobacteria Surveillance Study (CNTMS), including 76 strains of MAA and 27 strains of MAM (16). As shown in Table 1, we detected the MIC results of these strains and found that there were AMK resistant strains (MAA: five strains, MIC =128 μg/mL; MAM: one strain, MIC = 64 μg/mL) in both groups of MABC subsets. It was worth noting that there were induced drug-resistant strains (MAA: one strain, MIC = 16 μg/mL; MAM: one strain, MIC = 32 μg/mL) in both groups of MABC subsets after being treated with CLA for 3 days. In addition, after further treatment of CLA to 14 days, there was a significant difference in the rate of CLA resistant strains between the two MABC subsets. In the MAA group, 54.67% of the strains showed CLA resistance, but this was not found in the MAM group. Generally, there were far more CLA resistant strains than AMK resistant strains, which was consistent with previous reports (17). We further analyzed the different MIC results of the two groups of MABC subsets.

**TABLE 1:** AMK and CLA resistance of all MABC isolates^*a*^

Genotype	Drug	MIC range (μg/mL)	MIC50	MIC90	No. of strains distributed at the MIC (μg/mL)														
					0.015	0.03	0.06	0.12	0.25	0.5	1	2	4	8	16	32	64	128	256
MAA	AMK	1–128	2	32	0	0	0	0	0	0	26	16	10	8	6	5	0	**5**	0
	CLA (Day 3)	0.06–16	0.12	1	0	0	25	17	14	10	6	3	0	0	**1** ^*b*^	0	0	0	0
	CLA (Day 14)	0.06–32	8	32	0	0	5	6	6	8	5	4	**3**	**6**	**13**	**19**	0	0	0
MAM	AMK	1–64	2	16	0	0	0	0	0	0	8	6	4	3	4	1	**1**	0	0
	CLA (Day 3)	0.06–32	0.12	0.25	0	0	10	7	6	2	1	0	0	0	0	**1** ^*b*^	0	0	0
	CLA (Day 14)	0.06–2	0.25	0.5	0	0	3	7	10	3	2	1	0	0	0	0	0	0	0
Total	AMK	1–128	2	32	0	0	0	0	0	0	34	22	14	11	10	6	**1**	**5**	0
	CLA (Day 3)	0.06–32	0.12	0.5	0	0	35	24	20	12	7	3	0	0	**1** ^*b*^	**1** ^*b*^	0	0	0
	CLA (Day 14)	0.06–32	1	32	0	0	8	13	16	11	7	5	**3**	**6**	**13**	**19**	0	0	0

a CLA = clarithromycin; AMK = amikacin; MABC = Mycobacterium abscessus complex; MAA = M. abscessus subsp. abscessus; MAM = M. abscessus subsp. massiliense. Bold font indicates the drug-resistant strain.

b Indicates inducible resistance.

### Genotypes conferring clarithromycin and amikacin resistance.

**FIG 2:**
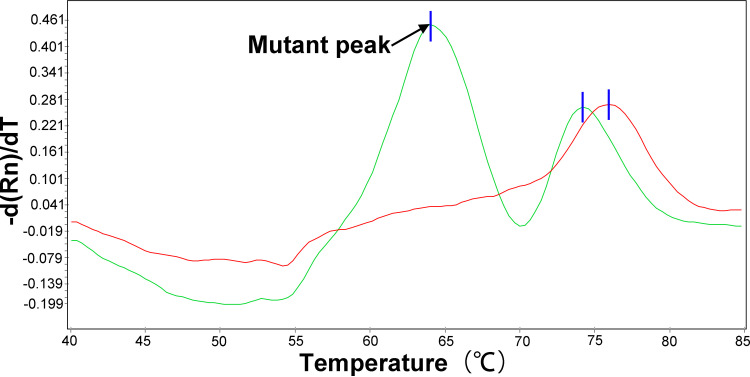
The melting curve chromatograms of CLA heteroresistant MAM isolate. The representative melting curve analysis of one MAA isolate (green) MeltPro MAB assay reveals that the mutant T allele of *erm*(41) locus in was mixed with the wild-type C allele relative to a positive control (red). Black arrows point to detectable mutant melting peak.

A large number of studies have shown that the specific genetic changes of *rrl*, *erm*(41) and *rrs* of MABC (*rrl*: A2270T; *erm*(41): T28, C28, Truncated; *rrs*: T1373N, A1373N, C1376N) were related to the production of CLA or AMK resistance (10–13, 18, 19). The results were shown in Table 2; one CLA resistant but AMK sensitive strain in both MAA and MAM groups harbored mutations in the *rrl* gene at position 2270 (A2270T), but the two strains showed different *erm*(41) gene phenotypes. Studies have confirmed that when the 28th nucleotide of the *erm*(41) gene is thymidine [*erm*(41)T28], the strain could induce methylated ribosomal 23s mRNA produced by the *erm*(41)T28 gene through CLA, thus preventing CLA from binding to the ribosome to produce inducible resistance (12). In addition, when the 28th nucleotide of *erm*(41) gene was cytidine [*erm*(41)C28] and truncated of *erm*(41), it would lead to the loss of gene function and produce CLA sensitivity (13). This study further confirmed this phenomenon through the verification of MeltPro MAB assay technology, 95.45% (42/44) of *erm*(41)T28 strains showed resistance to CLA, whereas two (4.55%) MAA isolates with *erm*(41)T28 genotype exhibited susceptible to CLA. Notably, we found 3 out of 44 isolates had two melting curve peaks (Fig. 2), representing the simultaneous presence of mutant and the wild type in these specimens. All *erm*(41)T28 strains showed CLA sensitivity, and 96.30% (26/27) of *erm*(41) truncated strains also showed CLA sensitivity. In addition, the mechanism of AMK resistance in MABC seems to be caused by point mutations of *rrs* specific genes (T1373A, A1375G, and C1376T; Escherichia coli numbering: T1406A, A1408G, and C1409T, respectively) (10, 18). However, this phenomenon was not found in this study.

**TABLE 2:** Summary of MABC genotypes and subspecies identification^*a*^

Genotype	CLA	AMK	No. of isolate	*rrl*	*erm*(41)^*b*^			*rrs*		
				A2270T	T28	C28	Truncated	T1373N	A1375G	C1376N
MAA	S	S	34	0	2	32	0	0	0	0
	R	S	37	1	37	0	0	0	0	0
	R	R	5	0	5	0	0	0	0	0
MAM	S	S	25	0	0	0	25	0	0	0
	R	S	1	1	0	0	1	0	0	0
	S	R	1	0	0	0	1	0	0	0

a CLA = clarithromycin; AMK = amikacin; MABC = Mycobacterium abscessus complex; MAA = M. abscessus subsp. abscessus; MAM = M. abscessus subsp. massiliense; R = resistant; S = susceptible.

b The results are obtained by MeltPro MAB assay.

### Performance of high-resolution melting curve analysis.

We then compared the CLA and AMK genotypic prediction and phenotypic susceptibility results in MABC and evaluated the prediction model. As shown in Table 3, the results for CLA showed high accuracy, specificity, and Kappa value (98.06%, 96.67%, and 0.96), and even sensitivity and negative predictive value (NPV) reached 100%. However, due to the limited number of cases of MABC, the Kappa value and positive predictive value (PPV) of AMK could not be detected in this study. This still requires us to further include more strains for research. Interestingly, our sequencing results on *rrs* showed that seven strains of MABC had three different point mutations (T558C, A976G, C977T), but all of them showed AMK sensitivity (2/97, 2.06%; 3/97, 3.09%; 2/97, 2.06%), which did not lead to AMK resistance (Table 4).

**TABLE 3:** Evaluation of CLA and AMK genotypic prediction versus phenotypic susceptibility results in MABC^*a*^

Genotypic prediction	Phenotypic susceptibility		Accuracy(95% CI）	Sensitivity(95% CI）	Specificity(95% CI）	PPV (95% CI)	NPV (95% CI)	Kappa value
	Resistant	Susceptible						
CLA			98.06% (95.70% to 100.00%)	100.00% (89.79% to 100.00%)	96.67% (87.45% to 99.42%)	95.56% (83.64% to 99.23%)	100.00% (92.26% to 100.00%)	0.96
Resistant	43	2^*b*^						
Susceptible	0	58						
AMK			94.17% (26.10% to 73.90%)	0.00% (0.00% to 48.32%)	100.00% (95.25% to 100%)		94.17% (87.25% to 97.61%)	0.00
Resistant	0	0						
Susceptible	6	97						

a CLA = clarithromycin; AMK = amikacin; MABC = Mycobacterium abscessus complex; PPV = positive predictive value; NPV = negative predictive value, CI = confidence interval.

b Two MAA isolates carrying *erm*(41) T28 fail to exhibit inducible CLA resistance.

**TABLE 4:** Mutations of the *rrs* gene among 103 MABC clinical isolates stratified to AMK susceptibility^*a*^

Mutation in *rrs*	No. of MABC isolates (%)	
	AMK-susceptible (*n* = 97)	AMK-resistant (*n* = 6)
T558C	2 (2.06%)	0 (0)
A976G	3 (3.09%)	0 (0)
C977T	2 (2.06%)	0 (0)

a AMK = amikacin; MABC = Mycobacterium abscessus complex.

## DISCUSSION

Due to the broad spectrum of drug resistance against many antibiotics, M. abscessus is likely to be difficult to treat medically (8). The *in vitro* susceptibilities to antimicrobial agents for clinical MABC isolates are recommended to form the effective chemotherapy regimens for MABC infection, although the correlation between the *in vitro* susceptibility of MABC isolates and the clinical outcome of corresponding antibiotics has not been established. Based on the ATS/IDSA guidelines, CLA, AMK, and cefoxitin are recommended for treating patients infected with MABC (20). In the present study, we demonstrated that MeltPro was a promising diagnostic for early detection of CLA resistance for MABC. There were increasing evidence that demonstrated a good correlation between phenotypic CLA resistance and mutations within *rrl* and *erm*(41) in MABC (14). In a recent cohort study, using the whole genome sequencing (WGS) assay, the sensitivity of existing knowledge for predicting CLA resistance was 95% (14). Compared with the WGS assay, which requires 3 to 5 days to complete sequencing and bioinformatics analysis, MeltPro can significantly improve the turnaround time for a CLA susceptibility result within 2 h (with no requirement for expensive instrumentation and easily interpreted). In addition, although WGS is able to identify additional variants outside the hot spot region conferring CLA resistance, this advantage is offset by its relatively high cost, which elevated diagnostic costs per patient with MABC infection by 10-fold ($500 for WGS versus $50 for MeltPro). Specially, the low cost of reagents is another obvious advantage of this assay considering that there is a clear link between lower socio-economic status and NTM infection (21). Thus, our data emphasizes the potential value of MeltPro assay as a promising diagnostic for CLA susceptibility in MABC.

Heteroresistance has been considered as a precursor stage for the emergence of drug resistance (22, 23). Numerous observational studies have demonstrated that the presence of a minor subpopulation of resistant cells causes poor treatment outcomes to antibiotic therapy (24, 25). The fact that heteroresistance is often undetected by conventional molecular assays highlights that it could be a significant cause of diagnostic inconsistency between genotypic and phenotypic methods. In our experiments, we also observed the coexistence of CLA resistant and CLA susceptible subpopulation in the MABC isolate, which may be potentially missed and diagnosed as CLA susceptible. We must acknowledge that the prevalence of CLA heteroresistance may be underestimated due to the inclusion of patients without macrolide exposure, and the dramatically increased incidence of CLA heteroresistance among MABC patients under treatment can be expected. Recent work indicates that a high-resolution melting curve outperforms conventional PCR-based assays for identifying heteroresistant samples (26). Thus, MeltPro is more likely to produce a better solution to accurate heteroresistance in MABC.

CLA resistance is a promising predictor of clinical outcomes for patients infected with MABC. In this study, we found that among 103 MABC isolates, 41.75% were resistant to CLA by 23S rRNA mutation or its methylation, indicating the elevated potential of treatment failure among MABC patients receiving CLA-containing regimens. The high proportion of CLA resistance emphasizes the urgent need for early detection of CLA resistance prior to empirical treatment of MABC infections. At least, subspecies identification should be conducted to differentiate M. abscessus and *M. massiliense* in view of significant difference in inducible CLA resistance between two major subspecies.

Another novel finding of our observation was that two M. abscessus isolates carrying *erm*(41)T28 failed to exhibit inducible CLA resistance. Although the exact mechanism remains unclear, we speculate that this contradictory result may be associated with impaired expression of the functional *erm*(41) methylase. We attempt to provide several plausible mechanisms for the loss of inducible resistance to CLA. On the one hand, a critical step in gene expression in the initiation of transcription at the core promoter. The sequence variation within core promoter regions substantially affects expression level of corresponding gene, which may be a partial explanation for this contradictory finding. On the other hand, the increases in *erm*(41) RNA levels after CLA exposure indicate the involvement of an inducible transcription factor (13). The malfunctioning effector for macrolides in the external environment is another possible explanation. Further experiments are needed to delineate its molecular mechanisms.

Multiple genetic variants of resistance in the 16S rRNA locus have been reported to be associated with AMK resistance (10). Previous studies demonstrated that three points mutations of 16S rRNA gene *rrs* (T1373N, A1375G, C1376N) could lead to a high level of AMK resistance. Several pervious have demonstrated that most of the AMK-resistant MABC isolates harbored a target site *rrs* mutation. In contrast, Lee and co-researchers found that *rrs* mutations were only present in one tenth of AMK-resistant MABC isolates from Korea (27). Our results were consistent with the latter situation, potentially reflecting that *rrs* mutations conferring AMK resistance may differ from one geographic region to another. Of note, we found that these 7 MABC strains harbored three different *rrs* point mutations, consisting of T558C, A976G, C977T, whereas they were also identified in AMK-susceptible isolates, affirming their role as phylogenetic SNPs rather than markers for AMK resistance, which was in consistent to the previous researches (10, 18). Interestingly, a study by Islam and colleagues revealed that mutations in the genes for 16S rRNA were uncommon in aminoglycoside-resistant Pseudomonas aeruginosa isolates (28). Therefore, we hypothesize that the mechanisms involve intrinsic drug resistance may be the main mechanism responsible for resistance to aminoglycosides in MABC circulating in China, such as the low permeability of the cell wall and induction of drug efflux pumps (6).

Although high accuracy and sensitivity have been achieved between CLA genotypic prediction and phenotypic susceptibility results in MABC in this study, we acknowledged that the major limitation of this study was the small number of MABC isolates due to the low incidence of this disease in China. This weakness would undoubtedly weaken the significance of our conclusion. Further multicenter prospective study is urgently required to validate our findings by enrollment of a large number of cases.

To conclude, this study firstly demonstrates that MeltPro is a promising diagnostic for early detection of CLA resistance for MABC isolates, which significantly improve the turnaround time within 2 h. Approximate two fifths of MABC isolates are resistant to CLA by 23S rRNA mutation or its methylation, emphasizing the urgent need for early detection of CLA resistance prior to empirical treatment of MABC infections. In addition, two MAA isolates carrying *erm*(41)T28 fail to exhibit inducible CLA resistance. Further experiments are needed to delineate its molecular mechanisms.

## MATERIALS AND METHODS

### Study population and strains.

In China, the National Surveillance of Nontuberculous Mycobacterium was conducted in 2019 (16). Species identification was performed using the MeltPro Myco assay, which can correctly differentiate 51 mycobacterial species (Zeesan Biotech, Xiamen, China). The subsequent partial gene sequencing of *rpoB* and *hsp65* were used to confirm the species identification of MABC as previously described (29). Prior to *in vitro* drug susceptibility testing, the strains were cultured on the Löwenstein-Jensen (L-J) medium at 37°C until a visible colony was formed. This study was approved by the Ethics Committee of Beijing Chest Hospital, Capital Medical University. The DNA sequences of *rrl*, *erm*(41) and *rrs* in this study have been submitted to GenBank.

### Minimal inhibition concentration.

Antimicrobial susceptibility testing was performed using commercially available plates for rapidly growing NTM on basis of strain identification (SensiTitre RAPMYCOI, Thermo Fisher). Briefly, the fresh colonies were harvested from the surface of L-J slants to saline with 0.02% Tween 80. The bacteria suspension was mixed vigorously on a vortex for 1 min until the bacterial colonies were dispersed homogeneously and then diluted to a 0.5 McFarland standard density. The inoculum was prepared by a 1:100 dilution of 0.5 McFarland standard MAB suspension in cation-adjusted Mueller-Hinton broth, and added to SensiTitre plates at 100 μL per well. The MIC range was 0.06 to 32 μg/mL for CLA, and 1 to 128 μg/mL for AMK. SensiTitre plates inoculated with rapidly growing mycobacteria were kept for 3 days. For CLA, the extended incubation (14 days) was performed for the detection of inducible resistance. Plates were read using the Vizion (Thermo Fisher). The breakpoints for interpretation of susceptibilities followed the Clinical and Laboratory Standards Institute (CLSI) guidelines, with 8 μg/mL for CLA and 64 μg/mL for AMK (30). The reference Mycobacterium peregrinum strain (ATCC 700686) was included in each batch experiment for quality control purposes.

### MeltPro MAB assay.

MeltPro MAB assay was run in a Slan-96S/P real-time PCR system (Zeesan Biotech) and conducted following the manufacturer’s instructions. Briefly, the genomic DNA was purified with freshly cultured colonies with the CTAB method. Before sample preparation, the genomic DNA was diluted at 1:20, which was used as a template for PCR amplification. Then, 5 μL of the genomic DNA was added to a reaction containing 20 μL of PCR mixture for each sample. The running program included decontamination at 50°C for 2 min, denaturation at 95°C for 10 min, 50 cycles of 95°C for 10 s, 57°C for 20 s, and 72°C for 30 s, followed by denaturation at 95°C for 2 min, hybridization at 35°C for 2 min, and temperature increase from 40°C to 85°C at a ramp rate of 0.04°C/s. The design of fluorescent probes for the selective detection of genotypes conferring CLA and AMK resistance were summarized in Fig. 1. Fluorescence was recorded at 6-carboxyfluorescein (FAM), HEX, ROX, and CY5 channels, which represented *rrl*, truncated *erm*(41), T/C genotype of *erm*(41) and *rrs* locus, respectively. Melting curve was obtained by plotting the negative derivative of fluorescence with respect to temperature versus temperature (dF/dT), and the Tm values were obtained by identifying the peaks of the melting curves. When completed, results regarding species were automatically provided by dedicated software (MeltPro Manager version 1.0; Zeesan Biotech) according to the result interpretation guidelines.

### Data analysis.

Results of phenotypical drug susceptibility testing were used as the reference standard to calculate the accuracy, sensitivity, specificity, positive predictive value (PPV), and negative predictive value (NPV) of the MeltPro MAB assay. The Kappa consistency test was used to evaluate the consistency between phenotypic and genotypic DST methods. Values of ≥0.75 indicate excellent agreement; values of 0.40 to 0.75 refer to moderate agreement; and values of <0.4 indicate poor agreement. All the calculations were done using SPSS 22.0 software.

### Ethics statement.

This study was approved by the Ethics Committee of Beijing Chest Hospital, Capital Medical University. Informed consent from all participating subjects was obtained.

### Data availability.

The data sets presented in this study can be found in online repositories. The names of the repository/repositories and accession number(s) can be found below: GenBank, OL704521-OL704623, OL704624-OL704726, ON194388-ON194490, ON194565-ON194667, ON221589-ON221664, ON221665-ON221691.
